# Location First: Targeting Acute Myeloid Leukemia Within Its Niche

**DOI:** 10.3390/jcm9051513

**Published:** 2020-05-18

**Authors:** Alice Pievani, Marta Biondi, Chiara Tomasoni, Andrea Biondi, Marta Serafini

**Affiliations:** 1 Centro Ricerca M. Tettamanti, Department of Pediatrics, University of Milano-Bicocca, 20900 Monza, Italy; alice.pievani@unimib.it (A.P.); m.biondi6@campus.unimib.it (M.B.); chiara94tomasoni@gmail.com (C.T.); 2 Department of Pediatrics, Pediatric Hematology-Oncology Unit, Fondazione MBBM/San Gerardo Hospital, 20900 Monza, Italy; abiondi.unimib@gmail.com

**Keywords:** acute myeloid leukemia (AML), leukemic stem cell (LSC), bone marrow stromal cells, bone marrow niche, targeted therapy

## Abstract

Despite extensive research and development of new treatments, acute myeloid leukemia (AML)-backbone therapy has remained essentially unchanged over the last decades and is frequently associated with poor outcomes. Eradicating the leukemic stem cells (LSCs) is the ultimate challenge in the treatment of AML. Emerging evidence suggests that AML remodels the bone marrow (BM) niche into a leukemia-permissive microenvironment while suppressing normal hematopoiesis. The mechanism of stromal-mediated protection of leukemic cells in the BM is complex and involves many adhesion molecules, chemokines, and cytokines. Targeting these factors may represent a valuable approach to complement existing therapies and overcome microenvironment-mediated drug resistance. Some strategies for dislodging LSCs and leukemic blasts from their protective niche have already been tested in patients and are in different phases of the process of clinical development. Other strategies, such as targeting the stromal cells remodeling processes, remain at pre-clinical stages. Development of humanized xenograft mouse models, which overcome the mismatch between human leukemia cells and the mouse BM niche, is required to generate physiologically relevant, patient-specific human niches in mice that can be used to unravel the role of human AML microenvironment and to carry out preclinical studies for the development of new targeted therapies.

## 1. Introduction

Acute myeloid leukemia (AML) is a hematologic cancer characterized by the abnormal clonal proliferation of undifferentiated blasts and by their primary infiltration of the hematopoietic organs, such as bone marrow (BM), lymph nodes and spleen. Uncontrolled expansion of leukemic cells suppresses normal hematopoiesis, leading to life-threatening thrombocytopenia, anemia, and immunodeficiency [1]. The AML-backbone treatment has not changed in the last 30 years, consisting of sequential cycles of chemotherapy with cytarabine (Ara-C) and daunorubicin [2]. Allogeneic hematopoietic stem cell transplantation (HSCT) represents the most effective consolidation option for intermediate- and high-risk AML patients [3]. However, disease relapse and progression remain the major causes of treatment failure [4]. Interactions between AML blasts and the BM microenvironment contribute to treatment failure as the niche can provide a sanctuary to AML cells and protect them from chemotherapy [5].

In the last decades, the identification of AML driver mutations such as FLT3 and IDH mutations and the development of specific target therapies have been associated with improved outcomes in this subset of patients [6,7,8]. Despite these recent advancements, the treatment of AML remains a significant unmet clinical need, especially for patients lacking targetable driver mutations. Moreover, AML is an extremely heterogeneous disease, with a complex genetic and cytogenetic landscape and a sub-clonal composition [9]. After therapy administration, resistant leukemic clones can be selected and cause disease recurrence [10]. Thus, approaches that target specific mutations in AML cells may result in the eradication of single subclones and, as a result, may be inadequate.

Therefore, targeting the altered BM microenvironment may represent a more useful approach for overcoming these limitations. Moreover, this strategy could be applied together with chemotherapy to a broad range of patients with various subtypes of disease and driver mutations as interactions between AML and their BM niche are not clone specific. Recent discoveries have identified a series of AML niche-specific features that could be targeted to suppress the self-reinforcing leukemic BM microenvironment and restore normal hematopoiesis. Some approaches for uncoupling leukemic stem cells (LSCs) and leukemic blasts from their protective niche have already been tested in patients and are in different phases of the process of clinical development. Other strategies, like targeting the mesenchymal stromal cells (MSCs) remodeling processes, are very promising but remain mainly at pre-clinical stages.

This review summarizes the recent advances in elucidating the AML BM stromal niche characteristics and point out new perspectives for targeting AML-niche interactions to contribute to the improvement of current treatments.

## 2. Interactions Between AML Cells and the Protective BM Stromal Niche

The BM microenvironment interacts with normal hematopoietic stem cells (HSCs) and leukemic cells in several ways affecting their cellular functions, including trafficking, adhesion, proliferation, differentiation, and quiescence. AML cells exploit stromal-dependent pro-survival signals and shape the BM microenvironment to create a permissive/self-reinforcing niche favorable for the maintenance and progression of chemotherapy-resistant AML, while suppressing normal hematopoiesis. The mechanism of stromal-mediated protection of leukemic cells and their anchorage to the BM is complex and involves many adhesion molecules, chemokines, and cytokines.

### 2.1. Adhesion of AML Cells to Their Niche

**Figure 1:**
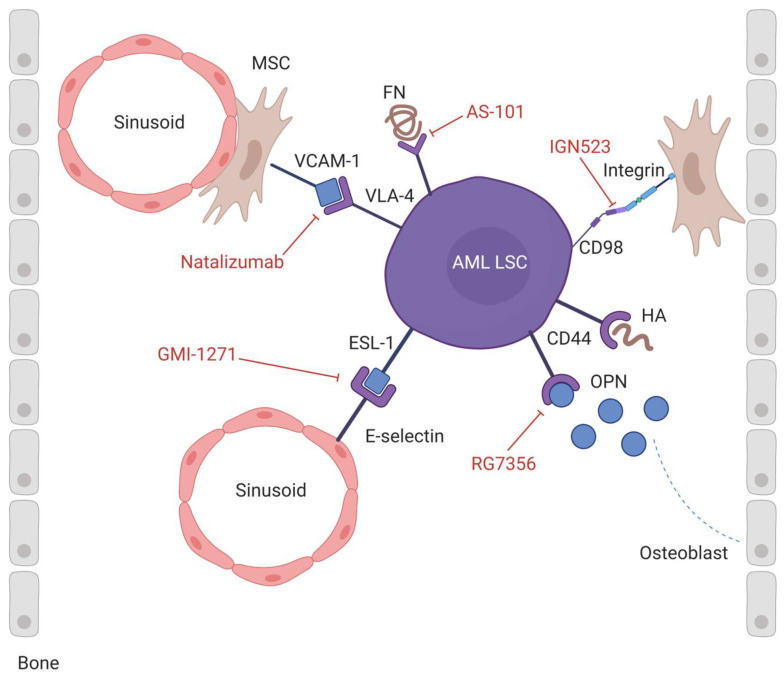
Acute myeloid leukemia (AML) cells interactions with the bone marrow (BM) niche. The BM microenvironment is composed of multiple different cell populations (mesenchymal stromal cells, adventitial reticular cells, sinusoidal endothelial cells, osteoblasts) and non-living extracellular matrix (osteopontin, fibronectin, hyaluronan). All these factors facilitate adhesion of LSCs and AML cells to the BM niche and regulate the migration, homing, survival, proliferation and chemotherapeutic agents’ resistance of AML cells. The following interactions have been reported to be involved: VLA-4/VCAM-1, VLA-4/FN, CD98/integrins, CD44/OPN, CD44/HA, ESL-1/E-selectin. All of them are under clinical investigation for therapies which may specifically disrupt the crosstalk of LSCs with the BM niche. The most relevant therapeutic molecules are outlined (red). MSC: mesenchymal stromal cell; LSC: leukemic stem cell; OPN: osteopontin; FN: fibronectin; HA: hyaluronan; VLA-4: very late antigen-4; VCAM-1: vascular cell adhesion molecule-1; ESL-1: E-selectin ligand-1. *This figure has been created with Biorender.com.*

AML cells hijack the BM niche by upregulating receptors on their cell surface, such as very late antigen-4 (VLA-4), CD44, E-selectin ligand-1 (ESL-1), and CD98, resulting in the retention of LSCs in the BM niche via interactions with adhesion molecules, such as vascular cell adhesion molecule-1 (VCAM-1), fibronectin (FN), hyaluronan (HA), osteopontin (OPN), selectins, and integrins. All these molecular interactions are potential targets for therapy since retention of LSCs in the BM niche results in quiescence and survival of LSCs and subsequently resistance to chemotherapy (Figure 1).

#### 2.1.1. VLA-4/VCAM-1-FN

Very late antigen-4 (VLA-4) is a cell surface adhesion molecule, composed of CD49d (α4) and CD29 (β1) subunits, which belongs to the integrin family [11]. VLA-4 is expressed by HSCs and hematopoietic progenitor cells (HPCs) and it plays an important role in the regulation of several physiological processes. In particular, the binding of VLA-4 with vascular cell adhesion molecule-1 (VCAM-1), expressed by MSCs, osteoblasts (OBs) and endothelial cells, or with FN, an extracellular matrix component, mediates homing and retention of HSCs [12]. Moreover, VLA-4 is constitutively expressed on most leukocytes where it takes part in immune cells recruitment to inflammation sites and antigen-presenting cell-lymphocytes interaction [13].

Jacamo et al. showed that VLA-4 is upregulated in AML cells and the interaction with VCAM-1 on stromal cells activates pro-survival and proliferative pathways in both leukemia and stromal cells via the nuclear factor-κB (NF-κB) pathway and favors chemoresistance. Interestingly, blockade of stromal NF-κB signaling can make AML cells more susceptible to chemotherapy [14]. Moreover, the interaction between VLA-4 on AML cells and FN on stromal cells results in the activation of the phosphoinositide-3-kinase (PI3K)/protein kinase B (Akt)/Bcl-2 signaling pathway and, ultimately, resistance to chemotherapy-induced apoptosis. Specifically, combined treatment in AML mouse models with anti-VLA-4 antibodies and Ara-C improves survival, and patients with VLA-4–negative AML have a more favorable prognosis [15]. Thus, the interaction between VLA-4 on leukemic cells and VCAM-1 or FN on stromal cells represents a promising candidate for targeted therapy.

Natalizumab is a humanized anti-VLA-4 monoclonal antibody (mAb) used for the treatment of autoimmune diseases, which causes prolonged HSC mobilization [16]. In a xenograft murine AML model, animals treated with Natalizumab had improved survival compared with control mice [17]. The molecular mechanism of this drug consists of the disruption of the interaction between VLA-4 and VCAM-1 through the binding of Natalizumab to the VLA-4 α4 subunit, inducing AML cell mobilization and chemosensitivity [18]. Despite its evident beneficial effects on overall survival, its utility is limited because it can induce leukoencephalopathy [19].

Another VLA-4 inhibitor is the synthetic tellurium compound AS-101 (ammonium trichloro(dioxoethylene-0,0’)tellurate), which induces redox inhibition of adjacent thiols in the exofacial domain of VLA-4 after binding to stromal FN. The inactivation produces cytoskeletal conformational changes that decrease the PI3K/Akt/Bcl-2 signaling [20] resulting in several biologic effects, such as inhibition of interleukin (IL)-10 and increase of AML cells chemosensitivity. Moreover, in a mouse xenograft AML model it has been shown that AS-101 can abrogate drug resistance of leukemic cells and prolong survival in mice after chemotherapy with Ara-C [20]. A clinical trial was registered (NCT01010373) to investigate the efficacy of AS-101 in combination with chemotherapy for elderly patients affected by AML and myelodysplastic syndrome, but it has been suspended.

#### 2.1.2. CD44/HA-OPN-Selectin

CD44 is a ubiquitously expressed transmembrane glycoprotein that is alternatively spliced, leading to the production of multiple protein isoforms. In the hematopoietic system, CD44 is expressed by hematopoietic precursors and regulates several functions as well as trafficking, lodgment, proliferation, apoptosis, and differentiation by binding to different ligands as HA, OPN, FN, and selectin [21,22].

CD44 is overexpressed in AML blasts and the presence of certain splice variants is associated with poor prognosis [23]. LSCs are more dependent on CD44 for homing and engraftment to BM niche compared with normal HSCs. In 2006, the pivotal role of CD44 in the interaction between LSCs and their niche was elucidated, demonstrating that engraftment of human AML cells in immunodeficient mice was decreased after treatment with an activating antibody to CD44 [24]. Notably, a simultaneous report by Krause et al. showed a role of CD44 in homing and repopulation of chronic myeloid leukemia (CML) stem cells, suggesting that both acute and chronic forms of myeloid leukemia may use CD44 for adhesive interactions of blasts to the marrow niche [25]. Moreover, it has been shown that high levels of CD44 are important for AML induction or relapse in AML mouse models [26]. Thus, targeting of CD44 represents a novel strategy to push LSCs out of their niche.

The use of H90, an anti-CD44 mAb, broke the interaction between CD44 and HA and caused a marked reduction of the leukemic burden in non-obese diabetic/severe combined immunodeficiency (NOD-SCID) mice transplanted with primary AML cells. Leukemic cells obtained from primary mice treated with H90 did not engraft into the secondary recipient mice, demonstrating that the antibody directly targeted LSCs [24]. Several mechanisms have been proposed to explain this suppression, including the abrogation of LSCs homing to the supportive niche and the alteration of LSCs fate, suggesting that CD44 may be a key regulator of LSCs properties.

Different mechanisms have been hypothesized to explain the effect of anti-CD44 mAbs on AML cells, such as the induction of cell differentiation and inhibition of cell proliferation [27]. Specifically, CD44 ligation by the mAb A3B8 reduces proteolysis of the cyclin-dependent kinase inhibitor (CKI) p27 which favors its binding with cyclin E/Cdk2 and results in the inhibition of the E/Cdk2 kinase activity that is correlated with the transition from G1 to S phase [28]. This blockade of S-phase entry ultimately reduces the proliferation of AML cells [27]. Moreover, A3B8 mAb-mediated AML cell proliferation inhibition, as well as in some cases the induction of differentiation, is accompanied by a marked decrease in the phosphorylation of the mammalian target of rapamycin complexes (mTORC)1 and 2, which is strongly correlated with the inhibition of the PI3K/Akt pathway [29]. However, the effect of anti-CD44 mAb observed in xenotransplantation models has not been confirmed in clinical studies so far. The humanized anti-CD44 antibody RG7356 was tested in a phase I trial in patients with refractory/relapsed AML [30]. Only one out of 44 patients achieved a complete response with incomplete platelet recovery, one patient achieved a partial response, and one experienced stable disease with hematologic improvement. The limited clinical activity observed in the monotherapy setting led to a combination therapy approach using RG7356 in combination with standard cytotoxic agents.

Osteopontin (OPN) is another ligand of CD44, which consents anchorage of LSCs in the HSC niche. Thus, besides the CD44-HA interaction, anti-CD44 antibodies may target CD44-OPN interaction as well. OPN expression was increased in BM blasts and in BM serum of AML patients as compared with healthy controls and OPN overexpression was related to a poor prognosis [31]. Blockade of the OPN signaling increases the number of cycling cells, inhibits homing and induces apoptosis in blasts and LSCs. Furthermore, the combination of anti-OPN mAb with Ara-C chemotherapy into acute lymphoblastic leukemia (ALL) engrafted mice showed a higher effect on the reduction of leukemic burden compared with Ara-C alone [32].

#### 2.1.3. ESL-1/E-Selectin

E-selectin is a cell adhesion molecule constitutively expressed by BM endothelium where it plays a key role in HSC and HPC homing. In particular, the deletion of E-selectin from endothelial cells increased HSC quiescence and self-renewal, confirming that E-selectin supports HSC functions [33]. Moreover, at inflammation sites, this molecule is involved in the regulation of leukocytes rolling along the luminal surface of endothelial cells. E-selectin can be overexpressed by endothelial cells activated by inflammatory cytokines such as tumor necrosis factor α (TNF-α) and interleukin-1 (IL-1) [34].

E-selectin ligand-1 (ESL-1) is expressed not only by HSCs but also by AML cells, where it induces blasts adhesion to the vascular niche and Wnt signaling activation, favoring the survival of AML blasts and promoting cell-adhesion mediated drug resistance [35]. E-selectin is upregulated five- to ten-fold on BM endothelium in AML [36]. CD44, the main receptor for HA, is another ligand for E-selectin on HSCs and LSCs.

E-selectin can be inhibited by the small-molecule antagonist Uproleselan (GMI-1271), which favors the reduction of cell survival and the increase of chemosensitivity, showing a contraction in the leukemic burden in xenograft AML models treated with a combination of chemotherapy and this E-selectin antagonist [35]. Thus, clinical trials with GMI-1271 in combination with chemotherapy are currently ongoing.

#### 2.1.4. CD98/Integrins

CD98 is a heterodimeric protein constituted by a heavy chain (CD98hc encoded by SLC3A2) that is a type II single-pass transmembrane glycoprotein, disulfide-linked to a multipass light chain that can be any one of six amino acid transporters (LAT1, LAT2, y1LAT1, y1LAT2, xCT, or asc-1) [37]. The heavy chain binds to integrins mediating cell adhesion and its association with a light chain regulates essential amino acid transport, which contributes to cell survival and growth [38]. In the hematopoietic system, CD98 is implicated in B and T cells proliferation and activation.

In AML, CD98 enhances the interaction between blasts and stromal cells and promotes maintenance and proliferation of leukemic cells [39]. For this reason, CD98 represents a suitable target for AML treatment. Notably, CD98 deficiency increases the survival of the AML mice and treatment with an anti-CD98 antibody inhibits the growth of human AML cells in xenograft models [39]. Specifically, treatment with the humanized mAb designated IGN523 resulted in a decrease of CD98 expression causing the reduction of amino acid transport and Bcl-2 expression, while increasing the lysosomal membrane permeability. Furthermore, IGN523 induced antibody-dependent cellular cytotoxicity (ADCC) and complement-dependent cytotoxicity (CDC) [40]. Therefore, anti-CD98 mAb therapy could mediate an anti-leukemia effect through multiple mechanisms, such as a reduction of cell adhesion to the microenvironment, increased apoptosis and ADCC/CDC.

Although clinical translation of these approaches targeting AML cell adhesion within the BM niche is still in its infancy, emerging early phase clinical data indicate this approach may be beneficial in the adjuvant setting, boosting the effects of conventional treatments.

### 2.2. Soluble Factors Secreted by AML BM Stromal Niche

**Figure 2:**
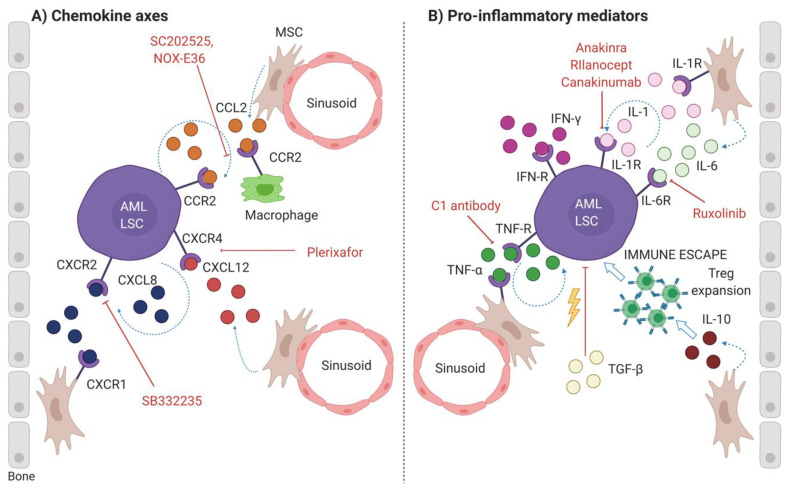
Soluble mediators involved in AML-BM niche crosstalk. (**A**) Chemokines contribute to cell proliferation, survival and chemotaxis. All these functions are important for the development of the AML-supportive BM niche. The chemokine axes involved in AML are the well-known CXCL12/CXCR4 axis and the newly investigated CCL2/CCR2 and CXCL8/CXCR1-CXCR2 axes. The most significant therapeutic agents and the pre-clinical molecules are outlined (red). At present, Plerixafor combined with conventional chemotherapy is under clinical investigation, whereas the other drugs represented refer to potential therapeutic approaches that still need to be evaluated in clinical trials. (**B**) In addition to chemokines, other subfamilies of cytokines are also dysregulated in the BM microenvironment. The pro-inflammatory mediators, such as interleukin (IL)-1, IL-6 and tumor necrosis factor (TNF)-α provide support to AML progression, while the immunosuppressive factors, like TGF-β and IL-10, can be downregulated/mutated and contribute to leukemia immune escape, respectively. Due to the pleiotropic nature of cytokines and the lack of detailed knowledge on the specific molecular players involved in their downstream signaling pathways, few clinical trials are investigating new drugs in the context of AML. The most promising are: Ruxolitinib, inhibiting IL-6/IL-6R interaction, that is being clinically evaluated in combination with decitabine in AML patients; Anakinra, Rilanocept and Canakinumab, which are FDA approved therapeutic agents indicated for inflammatory diseases, that still need to be tested in pre-clinical AML models. MSC: mesenchymal stromal cell; LSC: leukemic stem cell; CCL2: C-C motif chemokine ligand-2; CCR2: C-C motif chemokine receptor 2; CXCR1: C-X-C motif chemokine receptor 1; CXCR2: C-X-C motif chemokine receptor 2; CXCR4: C-X-C motif chemokine receptor 4; CXCL8: C-X-C motif chemokine ligand 8; CXCL12: C-X-C motif chemokine ligand 12; TNF- α: tumor necrosis factor alpha; TNF-R: tumor necrosis factor receptor; TGF-β: transforming growth factor beta; IFN-γ: interferon gamma; IFN-R: interferon receptor; IL-1: interleukin-1; IL-1R: interleukin-1 receptor. *This figure has been* *created with Biorender.com.*

Common chemokine axes, cytokine signaling pathways and pro-inflammatory or immunosuppressive mediators usually regulate homeostasis in the normal BM microenvironment. However, a series of studies suggest that these soluble factors may be hijacked by AML, promoting the maintenance of LSCs and, as a result, contributing to disease progression and recurrence [41] (Figure 2).

#### 2.2.1. Chemokine Axes

##### CXCL12-CXCR4 Axis

CXC motif ligand 12 (CXCL12), also known as stromal-derived factor 1α (SDF-1 α), is a chemokine mainly produced by MSCs in the adult BM [42]. The interaction between CXCL12 and its chemokine receptor 4 (CXCR4) plays a central role in HSCs maintenance, adhesion to the niche, survival and homing to the BM [43]. LSCs can compete with HSCs and exploit the CXCL12-CXCR4 axis to their advantage. Indeed, most AML blasts and especially LSCs acquire CXCR4 expression [44,45]. Moreover, high CXCR4 expression on AML is a negative prognostic factor associated with reduced overall and relapse-free survival [46]. CXCL12, similarly to its multi-functional effects on HSCs, acts as a pleiotropic chemokine also in AML. Mohle et al. first reported a role of CXCL12-CXCR4 axis in the regulation of AML cells homing and engraftment in the BM niche [47]. Moreover, CXCL12-CXCR4 contributes to AML chemoresistance, as it keeps leukemic cells in close contact with extracellular matrix components, like the integrin VLA-4 and the hyaluronate receptor CD44 [46], and with stromal cells that constitutively secrete growth-promoting and anti-apoptotic signals [48,49].

Considering the pathogenic role of CXCL12-CXCR4 axis in AML, much effort has been put toward finding strategies inducing its blockade [42,46]. Overall, four main classes of CXCR4 antagonists and agonists have been developed: (1) small peptide CXCR4 antagonists, like TN140 and its analogs [50], (2) non-peptide CXCR4 antagonists, such as Plerixafor (AMD3100), (3) mAbs against CXCR4, such as the fully human antibody Ulocuplumab [51,52], and finally, (4) modified agonist and antagonists for CXCL12 [53]. At present, CXCL12-CXCR4 inhibitors are explored in preclinical and clinical studies. Among those, clinical trials with Plerixafor are the most promising, as this CXCR4 antagonist efficiently mobilizes leukemic cells from BM to peripheral blood, thereby making them better targetable by conventional chemotherapy [54,55,56,57,58].

##### CCL2-CCR2 Axis

CC chemokine motif ligand 2 (CCL2), also called monocytes chemoattractant protein-1 (MCP-1), is a powerful chemoattractant for monocytes and macrophages to sites of inflammation. Upon binding to CC chemokine receptor 2 (CCR2), this chemokine mediates the activation of several intracellular pathways associated with survival, adhesion, proliferation, growth, chemotaxis and trans-endothelial migration [59]. Unlike the CXCL12-CXCR4 axis, whose role in AML has been clearly assessed, little is known about the CCL2-CCR2 axis. Recent evidence suggests that this chemokine axis is involved both in AML-BM niche interaction and in an autocrine loop in AML blasts. Regarding the former, several in vitro co-culture studies showed that BM-derived MSCs increase the secretion of CCL2 after contact with AML cells [14], but the functional impact of CCL2 in this context has not been deeply investigated yet. Regarding the latter, multiple studies display higher levels of CCR2 and CCL2 expression both in AML cell lines and primary AML samples compared to healthy controls [44,60,61,62]. However, Ramirez et al. observed that monocytoid AML patients show higher CCR2 expression, but significantly lower levels of CCL2 production compared to other AML subgroups and to normal controls [61], suggesting that the CCL2-CCR2 axis might be of particular relevance in this subset of patients. Macanas-Pirard et al. took a step further and demonstrated that the autocrine CCL2-CCR2 loop is involved in the regulation of chemotaxis and slightly improves proliferation of AML blasts, but does not contribute to chemoresistance [63]. On the contrary, Jacamo et al. suggests that the CCL2-CCR2 axis may be associated with AML resistance, not linked to its autocrine effects on AML blasts, but related to a subset of immunosuppressive macrophages in the bone. Their data show that CCL2-CCR2 inhibition in a mouse-to-mouse leukemia model is able to interfere with the infiltration of tumor-associated macrophages, especially in spleens of mice engrafted with leukemia.

In this scenario, blockage of CCL2-CCR2 interaction might increase chemotherapy efficacy by reducing the BM infiltration of M2-like macrophages, which normally downregulate effector cell types anti-tumor responses [62]. Overall, given the novelty of this concept, only pre-clinical studies have been conducted testing several CCL2-CCR2 inhibitors, like the synthetic CCR2 inhibitor SC202525, as well as mAbs directed against CCL2 and CCR2 [63], and NOX-E36, a human CCL2-specific RNA-like molecule [62]. Additional research is necessary to further understand the role of the CCL2-CCR2 axis in AML and its mechanism of action. However, to date the data suggest that this chemokine axis could represent a novel and promising target for the treatment of AML. Indeed, the beneficial effects of CCL2-CCR2 inhibition has been observed to impact multiple aspects of AML etiology as it reduces the migration and proliferation of AML cells and the recruitment of transformed macrophages that can take part in leukemia progression and resistance.

##### CXCL8-CXCR1/CXCR2 Axis

CXC chemokine ligand 8 (CXCL8), also known as IL-8, is a powerful pro-inflammatory chemokine involved in neutrophil chemotaxis and degranulation. This chemokine exerts its functions binding to two G-protein-coupled receptors, CXCR1 and CXCR2 [64]. High expression levels of CXCL8 and of its receptors have been extensively described in solid tumors. However, there is limited information about the role of CXCL8 in AML. A series of recent studies has indicated that leukemic blasts from AML patients overexpress the CXCL8-CXCR1/CXCR2 axis [44,64]. Moreover, the crosstalk between AML cells and the cellular components of the BM niche induces an increase in the local CXCL8 levels and the activation of downstream signaling pathways in these stromal cells [65,66]. All considered, AML-derived CXCL8 might be involved both in autocrine and paracrine loops in the BM niche. Concerning the autocrine circuit, Schinke et al. demonstrated that AML cells, especially LSCs, display increased CXCR2 expression and an augmented CXCL8 release. To discover the functional impact of this axis on AML progression, the authors inhibited CXCR2 by pharmacologic (adopting SB332235, a selective CXCR2-inhibitor) and genetic means and observed reduced proliferation and cell cycle arrest in the leukemia bulk. Additionally, CXCR2 inhibition decreased LSCs viability in vitro and enhanced leukemia mice survival in vivo. Besides, CXCR2 overexpression is associated with a worse prognosis, further highlighting the relevance of this axis in AML [64]. As previously mentioned, the CXCL8-CXCR1/CXCR2 axis is involved also in a paracrine circuit, acting as a mediator in the AML-BM niche network. According to Bruserud et al., the OBs present in the transformed BM microenvironment can contribute to leukemia progression, increasing AML cell proliferation and CXCL8 release [65]. Another work by Kuett et al. shows that CXCL8 production by AML cells can be influenced also by the hypoxic microenvironment. Indeed, AML blasts cultured in vitro for 48 h under severe hypoxic conditions (1% O_2_) display considerably higher levels of CXCL8 compared to those maintained under normal oxygen state (21% O_2_). Besides, CXCL8 released by AML cells interacts with CXCR1-expressing MSCs, determining their migration into the BM niche, where these stromal cells mediate chemoprotective, anti-apoptotic and pro-survival functions [66]. Additionally, Abdul-Aziz et al. showed that AML cells co-cultured with MSCs release macrophage inhibitory factor, which in turn induces CXCL8 synthesis by stromal cells, promoting AML survival [67].

Overall, these studies point out that CXCL8-CXCR1/CXCR2 might be a valid therapeutic target for the treatment of AML. Indeed, CXCR2 blockage may act selectively against LSCs, while sparing normal HSCs. Whereas, CXCR1 blockage may inhibit AML blasts-MSCs crosstalk, determining the loss of support signals for AML cells. By now, several drugs, like small molecule inhibitors, have been tested for the treatment of solid tumors [68], but in the context of AML, no clinical trials have been approved targeting CXCL8 or its receptors.

#### 2.2.2. Pro-Inflammatory Mediators

##### IL-1 Pathway

IL-1 family is composed of several factors, among which IL-1α and IL-1β are the most widely characterized. IL-1α is constitutively secreted by different cell types in homeostatic conditions and its production increases following an inflammatory stimulus. On the contrary, IL-1β is released by a restricted selection of cell types, primarily myeloid cells. Both cytokines interact with IL-1 receptor 1 (IL-1R1) and activate a downstream signaling cascade that mediates the transcription of several target genes [69]. Hence, IL-1 acts as a multifunctional factor, regulating a series of physiological functions. In particular, IL-1β plays a central role in the mediation of innate and adaptive immune responses, both local and systemic. Further, IL-1β is a crucial growth factor for MSCs and it is involved in hematopoiesis, enhancing stromal cells ability to maintain HSCs [69]. Several studies suggest that IL-1, especially IL-1β, is associated with AML pathogenesis. Indeed, elevated IL-1β serum levels have been reported in patients with leukemia and are associated with poor prognosis [70,71]. Due to its pleiotropic nature, IL-1β can influence leukemogenesis under several aspects and it can act both as an autocrine or paracrine factor. Specifically, IL-1β can promote AML blasts proliferation and survival, through the generation of a pro-inflammatory BM microenvironment, enhancing the production of other pro-leukemic chemokines and disrupting the anti-tumor immune response. Carter et al. tried to understand how IL-1β could be involved in the cross-talk between AML and MSCs and discovered that MSCs co-cultured with AML cells upregulate the apoptosis repressor with caspase recruitment domain (ARC), which induces IL-1β expression in AML blasts, that in turn augments the production of CCL2, CCL4, and CXCL12 by MSCs [72]. Furthermore, another study reported that IL-1β can increase the production of myeloid cell proliferation factors, like granulocyte-colony stimulating factor (G-CSF), and enhance AML cell proliferation [73]. Additionally, high IL-1β levels can contribute to leukemia progression, altering the anti-tumor immune response and contributing to myeloid-derived suppressor cell (MDSCs) generation [74]. Although these data expand our knowledge on the IL-1 pathway in AML, its role is still debated. Indeed, other studies report quite opposite results. For instance, Su et al. observed lower levels of IL-1β in patient-derived samples compared to normal controls [75]. Moreover, Yang et al. described that leukemia progenitor cells display lower levels of IL-1β expression in comparison with AML bulk and normal HSCs [76]. These contrasting data are likely justified by the different subtypes of AML included in the cohorts analyzed or the transformed cell population under study (LSCs or AML blasts). However, this highlights the need for more comprehensive research. All considered, the blockade of IL-1 signaling pathway might be a potentially effective strategy to treat AML.

A series of therapeutic agents have been approved by the FDA for the treatment of several chronic and inflammatory diseases, but they have not been tested yet in clinical trials for AML. These drugs can be classified as (1) natural or recombinant IL-1 inhibitors, like IL-1Ra and Anakinra (Kineret), (2) IL-1 decoy receptors, such as Rilanocept (Arcalyst) and (3) mAbs, as Canakinumab (Ilaris) [69]. A phase I clinical trial (NCT01260545) testing the IL-1α inhibitor, CA-18C3, was approved in 2010 for patients with advanced hematological malignancies, but its results are still not available. Undoubtedly, representative preclinical models of AML are necessary to deepen the study of the IL-1 pathway and develop new drugs specifically acting in this context. As of today, two mAbs directed against the IL-1 receptor accessory protein (IL1RAP) are being pre-clinically tested in AML. IL1RAP is a promising potential target due to its high expression on LSCs in most AML patients, but not on HSCs. CSC012-ADC, developed by Cellerant Therapeutics, is an antibody-drug conjugate (ADC) which has shown promising results both in vitro and in vivo, suggesting it might become a good targeted treatment for AML. Cantargia’s, Can-04, also known as Nidanilimab, is another mAb that mediates a powerful ADCC response, engaging natural killer (NK) cells against leukemia [77]. In 2015, Agerstam et al. published a study in collaboration with Cantargia, which demonstrated that Nidanilimab mediated a potent anti-tumor effect in xenograft models of primary human AML cells [78]. These promising pre-clinical results obtained in the hematologic space led to Nidanilimab also being tested in an open label phase I/IIa clinical trial for the treatment of patients with pancreatic cancer or non-small cell lung cancer. Taken together, Nidanilimab could serve as a beneficial immune-oncology agent for the treatment of multiple cancer types, AML included.

##### TNF-α Signaling

TNF-α is a pro-inflammatory cytokine and a pleiotropic factor, as it takes part in a wide range of functions, such as inflammatory responses, anti-tumor actions and homeostasis. TNF-α exerts these activities through binding tumor necrosis factor receptors (TNF-R)1 and 2. TNF-R1 is expressed by many different cell types and regulates cytotoxicity, resistance to infections and activation of the NF-κB pathway, whereas TNF-R2 expression is restricted to the hematopoietic lineage and it is activated only in inflammatory conditions. TNF-α role as a HSC growth factor regulator highly depends on the microenvironment which can determine both an inhibitory and a stimulatory effect on HSCs proliferation and maintenance [79]. TNF-α occurs in two different bioactive forms: the membrane-bound TNF-α (tmTNF-α) is cleaved by the TNF converting enzyme (TACE), generating the secretory form of TNF-α (sTNF-α) [80]. The role of TNF-α in AML is amply debated. For instance, a study by Sanchez-Correa reported that mean TNF-α expression was significantly increased in AML patients compared to healthy donors [81] and elevated serum levels of TNF-α may contribute to adverse prognosis in AML patients [82]. Comparatively, another study did not observe a high expression of TNF-α in primary AML samples [83]. Indeed, previous work reported that TNF-α contributes to anti-tumor activities, mediating both direct and indirect cytotoxic functions. However new evidence strongly suggests that TNF-α acts both as an autocrine and paracrine factor, promoting AML development and proliferation [80]. Concerning the autocrine function, TNF-α, in particular its membrane-bound form, promotes AML cell survival through the activation of the NF-κB and c-Jun N-terminal kinase (JNK)/activator protein-1 (AP-1) pathway, inducing the transcription of their anti-apoptotic target genes. Interestingly, these axes seem to be particularly active in LSCs, maintaining their survival and promoting AML relapses [84]. Regarding its role as a paracrine factor, it has been reported that each form of TNF-α mediates a different function [80]. sTNF-α is responsible for the development of a leukemia supportive niche and it can remodel BM-derived MSCs, increasing their migratory function and making them acquire a tumor supportive cancer-associated fibroblasts (CAFs) phenotype [85,86,87], whereas tmTNF-α is involved in the immunosuppressive activities of T regulatory cells (Tregs) and MDSCs, enhancing leukemia immune escape [88,89]. According to Zhou et al., tmTNF-α, due to its bifunctional role as an autocrine ad paracrine mediator, could be a better prognostic marker and a potential therapeutic target for AML treatment. To verify their hypothesis, the authors tested a tmTNF-α-specific mAb, called C1, on AML cells. They observed that leukemia cell chemosensitivity increased in vitro and AML engraftment was delayed in vivo, without causing HSCs toxicity [84]. Overall, both forms of TNF-α contribute to leukemia progression, but much work has to be done to deepen our understanding of the different mechanisms in which they are involved and to develop new specific anti-TNF-α therapies against leukemia.

##### IL-6 Pathway

IL-6 is a multifunctional cytokine released by several cell types, like innate and adaptive immune and stromal cells. This cytokine exerts its functions by binding to the IL-6 receptor (IL-6R/CD126). Primarily, IL-6 is a mediator of acute-phase and immune responses, but it plays a role also in cell proliferation, survival, differentiation and migration. Additionally, IL-6 is implicated in the regulation of normal hematopoiesis, and in malignant conditions it seems to be involved in AML blasts formation [90]. Accordingly, high levels of IL-6 in the plasma have been detected in multiple patients with preleukemic and leukemic diseases. Moreover, Reikvam et al. reported that primary AML cells co-cultured with healthy donor-derived MSCs induce an increment in IL-6 release into the media [91]. Furthermore, Lopes et al. showed that the increased amount of IL-6 produced by MSCs is directly correlated with disease progression from myelodysplastic syndrome (MDS) to AML [92]. In contrast to this study, Kittang et al. demonstrated that MDS patients display higher IL-6 production compared to healthy donors, but the authors did not find any correlation between IL-6 levels and progression to AML [93]. All things considered, the clinical impact of high IL-6 levels in AML is still not clear and its use as a predictive biomarker needs to be more deeply investigated. Adding further complexity, IL-6 seems to mediate divergent effects on AML blasts proliferation. Indeed, in some patient-derived samples IL-6 induced leukemic cell proliferation [75,94], while in others, AML blasts proliferation was either not affected or even diminished [95,96]. Even though the IL-6 role in AML is still not clear, high IL-6 levels have been definitively associated with chronic and autoimmune diseases.

For this reason, several strategies have been developed to block IL-6 or its receptor [90]. Essentially, they can be classified into three main classes: (1) mAbs such as Tocilizumab, (2) recombinant proteins, (3) small molecule inhibitors like Ruxolitinib. Among those, Ruxolitinib is the most interesting in the context of AML. This drug blocks JAK/STAT signaling activated by MSC-derived cytokines, such as IL-6, and is currently under a phase I/II clinical trial for post-myeloproliferative neoplasm secondary AML to evaluate its therapeutic efficacy in combination with decitabine (NCT03558607). The best overall response rate was 45% in the 18 patients treated with the established regimen. Unfortunately, the survival rate was poor, and the majority of patients died before allogeneic HSCT. However, considering their old age (mean = 69 years), this treatment protocol might be a safer alternative to intensive conventional chemotherapeutic agents while granting comparable response rates [97]. Further pre-clinical and clinical studies are necessary to test the potentialities of Ruxolitinib and find potentially synergic drugs.

##### IFN-α/β Signaling

Interferon alfa and beta (IFN-α/β) are type I interferons which exert their functions through the interaction with the IFN-α/β receptor. IFN-α and IFN-β are part of a composite group of factors that activate in response to viral antigens and stimulate anti-proliferative and immunomodulatory functions [79]. Multiple studies have proven the anti-leukemic activity mediated by type I interferons. In particular, IFN-α can mediate direct effects on AML blasts, limiting the release of leukemia supportive soluble factors, inhibiting their proliferation, inducing apoptosis and contributing to AML cell recognition by the immune system. Additionally, IFN-α can induce indirect effects on AML by activating dendritic cells (DCs) and adaptive immune cells, like T cells and NK cells, that are the most effective anti-tumor mediators [98]. Overall, the multiple anti-leukemic functions mediated by IFN-α supported its use in AML treatment. The proof of IFN-α efficacy as a therapy for AML first came in 1979 [99], and since then multiple clinical trials have been run. Over the years, the therapeutic action of IFN-α has been extensively studied, both as a monotherapy and in combination with other treatments. Mainly three distinct therapeutic settings have been tested: induction therapy, salvage therapy and post-remission treatment to prevent AML recurrence. However, even if IFN-α had the potential to mediate potent effects in AML treatment, the results obtained from these clinical trials were really discordant and not so encouraging. Several factors might have played a role in determining these divergent clinical outcomes: different study designs, inter-subject variability, different dose regimens; but above all, the key factor for IFN-α therapeutic efficacy seem to be its persistency in the serum of patients [100]. Hence, researchers developed long-acting IFN-α formulations, like IFN-α conjugated to a polyethylene glycol moiety (pegylated-IFN-α). These new preparations might avoid fluctuations in IFN-α plasma concentrations and are currently under evaluation [79,101].

##### IFN-γ Signaling

Interferon-gamma (IFN-γ) is a type II interferon and a pro-inflammatory cytokine produced by activated T lymphocytes and NK cells. It mediates multiple effects on a wide range of cell types, interacting with its receptors IFNGR1 and IFNGR2. Essentially, IFN-γ is a key mediator between innate and adaptive immune responses. It is involved in the regulation of many other immunomodulatory factors and it plays a role in HSCs regulation, stimulating their proliferation [41]. Accordingly, it was observed that chronic IFN-γ production can induce HSCs depletion and might lead to hematological diseases [102]. Specifically, excessive IFN-γ release has been observed in MDS patients and seems to be linked with disease progression and chemosensitivity [79]. However, contrary to the pro-tumorigenic function of IFN-γ in MDS, it seems that this pro-inflammatory cytokine may mediate the opposite effect in AML. For instance, Ersvaer et al. reported that IFN-γ, when combined with other specific factors such as IL-1β, granulocyte-macrophage colony-stimulating factor (GM-CSF), G-CSF and stem cell factor (SCF), significantly reduces AML blasts proliferation in vitro. Moreover, the authors observed that IFN-γ can reduce CXCL8 production and promote the synthesis of the anti-angiogenic chemokine, CXCL9-11. However, high levels of IFN-γ may have an opposite impact on AML cell apoptosis both alone and in combination with Ara-C in different patient-derived samples [103]. Overall, it seems that the effects mediated by IFN-γ are highly dependent on the surrounding cytokine network. Hence, even if these preliminary results suggest that IFN-γ mediates antileukemic activity, further pre-clinical studies and representative in vivo models are necessary to identify the proper clinical setting for IFN-γ targeting in AML [79].

#### 2.2.3. Immunosuppressive Cytokines and Others Mediators

##### TGF-β Signaling

Transforming growth factor β (TGF-β) superfamily is a group of 32 factors that includes TGF-β. The most investigated members of this family are the three different isoforms of TGF-β (TGF-β1, TGF-β2, and TGF-β3), especially TGF-β1, which is ubiquitously expressed and highly secreted [104]. TGF-β is synthesized as a pro-factor which requires proteolytical cleavage for biologic activity. Once activated, TGF-β isoforms interact with two receptors, the type I receptor (TβRI) and the type II receptor (TβRII), which are ubiquitously expressed. The downstream signaling activated by TGF-β is a classical membrane-to-nucleus pathway which involves the receptor-activated SMAD (small mothers against decapentaplegic) transcription factors that regulate the transcription of a wide range of TGF-β responsive genes. TGF-β pathway is in charge of multiple cellular functions, such as proliferation, differentiation, migration and survival, but also of many physiological processes, like hematopoiesis and immunity. During HSC differentiation, TGF-β acts as a negative mediator of proliferation, but at the same time it can stimulate cell division and apoptosis when it is necessary [105]. TGF-β plays a fundamental role also in the immune system, acting as an immunosuppressive factor blocking T cell effector functions and promoting Treg expansion to induce self-tolerance [106]. In the context of hematological disorders, such as AML, TGF-β can act both as a tumor suppressor, due to its role in cell proliferation and apoptosis, but also as a tumor promoter because of its effects on migration and downregulation of effective immune responses [106]. Concerning the first, leukemia is commonly characterized by the development of a TGF-β resistance mechanism which consists of the downregulation of TGF-β or its receptors, or by the mutation/deletion of the intracellular mediators of TGF-β signaling pathway, like the SMAD proteins. Accordingly, significantly decreased levels of TGF-β expression were detected in a large cohort of AML patients [81,107]. Additionally, TGF-β was found to promote apoptosis in AML patient-derived samples [71] and to inhibit the self-renewal ability of LSCs [108]. However, TGF-β may play also the opposite function, as high TGF-β levels can promote leukemia progression due to its interaction with the stroma and its immunosuppressive activity. For instance, Gey et al. reported that TGF-β is a trigger factor causing the development of functional and structural deficits in healthy MSCs, similar to those observed in AML patient-derived MSCs. These alterations are prevented by the TGF-β receptor I kinase inhibitor, SD-208, confirming that TGF-β plays a pivotal role in the AML BM microenvironment [109]. Nonetheless, it is still not clear how TGF-β functions are regulated in leukemia. Some hypotheses suggest that TGF-β may act as a tumor suppressor at the early stages of the disease, whereas it becomes a tumor promoter when the cancer is at its late stages [107].

Several attempts to target the TGF-β signaling pathway have been made in solid tumors. Three principal classes of drugs have been developed: (1) small molecule TβRI inhibitors, among which Galunisertib (LY2157299) is the most widely studied. This compound has shown little but consistent therapeutic efficacy in two phase II clinical trials for pancreatic cancer and hepatocellular carcinoma patients. (2) Antibodies directed against TGF-β, like 1D11, an anti-pan-TGF-β antibody and its humanized form, Fresolimumab, that are being tested for the treatment of multiple types of solid tumors, and lastly, (3) receptor-based TGF-β traps; AVID200, which selectively binds and inhibits the TGF-β1 isoform, is of particular interest since it is currently under phase I clinical trials for the treatment of advanced and metastatic solid tumors [104]. To obtain similar results in hematological malignancies, first it is necessary to go further in the definition of the specific molecular players involved in TGF-β signaling and its interactions.

##### IL-10 Pathway

IL-10 is an immunosuppressive multifunctional cytokine produced by several cell types, like Tregs, macrophages, B-cells and keratinocytes. This factor interacts with its two receptors, IL-10 receptor 1 (IL-10R1) and IL-10 receptor 2 (IL-10R2), to exert its pleiotropic effects. Specifically, IL-10 is involved in the inhibition of the effector functions of T lymphocytes, monocytes and macrophages and in the downregulation of pro-inflammatory cytokines to limit and eventually conclude inflammatory responses [41]. Like TGF-β, IL-10 also seems to play a dual role in AML. IL-10 may act as a tumor suppressor as it reduces the expression of pro-inflammatory cytokines supporting AML cell proliferation, but it can be also a tumor promoter due to its strong immunosuppressive activity, inhibiting the proliferation of effector T cells while boosting Tregs expansion. In support of IL-10 tumor suppressor action, several studies observed that IL-10 was able to induce AML blasts apoptosis in vitro probably through the inhibition of proinflammatory cytokines production, such as IL-1α, IL-1β, IL-6, GM-CSF and TNF-α [110,111,112]. Moreover, it has been reported that AML patients with high IL-10 plasma levels display better chemotherapy responses and superior survival rates [81]. Otherwise, IL-10 may operate also as a tumor promoter. For instance, recent research suggests a significant correlation between high levels of IL-10, produced by BM-derived MSCs, and reduced survival of AML patients. Additionally, the authors did not detect any differences in the levels of pro-inflammatory cytokines released by patient-derived MSCs and normal controls [113]. Furthermore, Wu et al. reported that Tregs can contribute to the increased IL-10 levels observed in AML. Patients with higher Treg cell frequency at diagnosis have a worse prognosis. Indeed, Tregs expanding in the peripheral blood of AML patients mediate immunosuppressive effects through IL-10 secretion, blocking IFN-γ and IL-2 production and effector T cells proliferation, potentially contributing leukemia immune escape [114].

##### VEGF Signaling

Vascular endothelial growth factor (VEGF) is a proangiogenic factor that binds to specific tyrosine kinase receptors on endothelial cells (EC) leading to the induction of angiogenesis. Neoangiogenesis has been shown to play an important role in the pathogenesis of AML and an increased number of ECs have been noted on BM biopsies of patients [115]. AML blasts in about 50% of patients constitutively secrete VEGF, which has been shown to represent a major inducer of proliferation and activation of ECs [116]. Activated ECs produce hematopoietic growth factors such as SCF or GM-CSF, which may be necessary for leukemic cell growth and survival in a paracrine fashion. AML blasts themselves, besides receptors for growth factors, express also VEGF receptors in about 10% to 20% of cases [116]. These receptors may be involved in leukemic cell proliferation and resistance to apoptosis by responding to growth stimuli provided by ECs of the niche and to autocrine factors [117]. VEGF-dependent EC activation increases also EC-AML cells adhesion and AML aggressiveness [118], and a high VEGF plasma concentration is associated with adverse prognosis [119]. ECs can also confer chemotherapy resistance to AML cells. One study observed that AML cells acquired EC-like features and integrated into the blood vessel where they can become quiescent and evade chemotherapeutic treatment [120].

AML may, therefore, represent a disease that is sensitive to a therapy directed against the effects of VEGF [121]. To date, several drugs with antiangiogenic properties had been tested in clinical studies, but clinical outcomes have been discouraging [122]. Targeting VEGF signaling with the recombinant humanized mAb Bevacizumab in AML has not been successful so far [123], but their combination with standard induction chemotherapy might be more promising [124]. Tyrosine kinase inhibitors (e.g., Semaxanib, Sunitinib, and Sorafenib) that bind to a broad range of receptor tyrosine kinases including VEGFR are under clinical investigation for AML treatment with relevance to angiogenesis. Both Semaxanib and Sunitinib showed only modest clinical activity in AML treatment [125,126]. Sorafenib monotherapy or in combination with conventional chemotherapy has shown a reduction of leukemic growth in AML patients carrying FLT3 mutations but not in patients without mutations [127]. Patient response to antiangiogenetic therapy is heterogeneous and identifying susceptible subgroups seems to be crucial.

### 2.3. Signaling via Mitochondrial Transfer in AML Niche

Beyond adhesion molecules and soluble factors, another mechanism has recently been ascribed to AML-niche interaction. Mitochondrial transfer is a newly described way of communication between BM-derived MSCs and AML cells, which consists of the delivery of fully functional mitochondria from the donor stromal cell to the recipient leukemic cell [128]. Given the novelty of this mechanism, basically two research groups have tried to assess the role of mitochondrial transfer in the AML microenvironment. The authors performed in vitro co-culture assays that revealed the intercellular horizontal transfer of mitochondria results in greater mitochondrial mass in leukemic cells compared to normal HSCs, contributing to increased ATP production through augmented oxidative phosphorylation (OXPHOS), reduced apoptosis and chemotherapy resistance [129,130,131]. In vivo experiments further confirmed the delivery of murine mitochondrial DNA to primary AML cells isolated from BM of immunodeficient NSG mice that had been injected with these cells [130,131]. Notably, it was observed that LSCs specifically rely on mitochondrial function for their survival and take advantage of this mechanism to maintain their energy generation ability and increase the apoptotic threshold [132].

The signaling pathways and the molecular processes involved in mitochondria uptake from AML cells are still not well characterized. However, the consensus in the available literature suggests that this process of transfer demands cell-to-cell contact. Indeed, Moschoi et al. observed, in a niche-like co-culture system, that mitochondrial transfer would occur only if MSCs and AML cells were in direct contact in the BM microenvironment. According to their model, mitochondrial uptake was mediated by endocytosis, but they were not able to define the players involved in this system [130]. Marlein et al. went a little further and provided a detailed description of the process regulating mitochondrial transfer. These authors have suggested that mitochondria uptake by leukemic cells occurs primarily through filamentous actin-based protrusions called tunneling nanotubes (TNTs) [128,131]. TNTs have been described both in cell lines and patient-derived AML cells as structures able to link different cells and to act as a track to carry organelles, such as mitochondria. Actually, the use of a fluorescent tracking system provided the demonstration that TNTs extend from MSCs toward AML cells [133]. Consistent with this assumption, cytochalasin B, an inhibitor of actin polymerization, prevented TNT formation and a significant reduction in the percentage of AML cells that acquire MSCs mitochondria was observed [131]. Subsequently, to identify the molecular players involved in mitochondrial transfer, Marlein et al. tried to uncover the trigger signals of this process. Through the screening of several pharmacological agents, the authors found that drugs like N-acetyl cysteine, diphenyleneiodonium (DPI) and glutathione reduce mitochondrial transfer, whereas hydrogen peroxide (H2O2) and doxo/daunorubicin increase it. These findings suggested that an altered redox state might be the stimulus behind mitochondrial transfer. Indeed, the co-culture of BM-derived MSCs with AML cells showed an increase in reactive oxygen species (ROS) release and oxidative stress in stromal cells. According to their model, AML cells, through NADPH oxidase-2 (NOX2) activity, locally increase oxidative stress, driving MSCs to produce new mitochondria. The fully functional mitochondria are then delivered to AML cells through TNTs without causing a metabolic imbalance of MSCs as they activate the peroxisome proliferator-activated receptor γ coactivator (PGC)-1α, the master regulator of mitochondrial biogenesis, which keeps ROS levels under control [134].

All things considered, mitochondrial transfer might be described as a mechanism employed by AML cells to rescue respiration, acquire a metabolic advantage and survive even after exposure to chemotherapy. Indeed, the majority of chemotherapeutics are based upon processes involving the induction of oxidative stress. Hence, leukemic cells with extra-functional mitochondria might survive after oxidative chemotherapy and eventually regenerate the disease.

Targeting mitochondrial transfer might be an appealing approach for the development of improved therapeutic strategies able to eradicate resistant AML cells. Considering that mitochondrial metabolism has emerged as a vulnerable point for LSCs, the inhibition of mitochondrial transfer might contribute to disease clearance [132]. Additionally, targeted therapeutic approaches might be feasible as normal HSCs are less likely to accept extra-mitochondria and thus have minimal toxic effects. While currently we are still at a very early stage, pre-clinical research is already testing new ideas. For instance, it may be possible to prevent the transfer of mitochondria to AML cells by treating recipient cells with antioxidants. Another strategy could be developing drugs targeting the mitochondria trafficking machinery and especially TNTs such as inhibitors of actin polymerization [129]. Otherwise, the two players involved in the mechanism at the basis of mitochondrial transfer, NOX2 and PGC-1α, might be evaluated as potential targets. Actually, NOX knockdown (NOX2 KD) via short hairpin RNA or pharmacological inhibition causes reduced ROS production in MSCs, decreased MSC-to-AML transfer of mitochondria and diminished maximum mitochondrial respiration in AML blasts. PGC-1α inhibition also affects mitochondria transport. Moreover, both molecules seem to be required for AML persistence and recurrence in vivo. Indeed, mice infused with NOX2 KD AML cell lines exhibit reduced engraftment and extended overall survival, while PGC-1α KD MSCs mice display a reduced tumor volume compared to control mice [131,134]. Overall, these data pinpoint some potential targetable antigens, but much work still has to be done.

### 2.4. Niche-Dependent Regulation of Hypoxia

Hypoxia is a key regulator of the niche-mediated quiescence and maintenance of HSCs [135,136]. Low oxygen supply has direct effects on the metabolism of a cell, shifting from OXPHOS to cytoplasmic glycolysis [137]. The latter is less efficient in energy production and forces cells to quiescence for energy conservation. A benefit of shutting down OXPHOS is the reduction of intracellular ROS levels produced. Decreased ROS are crucial to keep HSCs in a dormancy state since ROS are pivotal inducers of differentiation, proliferation, migration and depletion of the pool of quiescent HSCs.

AML cells are preferentially home to hypoxic niches where their exposure to drugs and immune effector cells is reduced through the compromised blood flow, and these BM areas contain true quiescent and chemoresistant LSCs endowed with a self-renewal capability [138]. Hypoxia activates the hypoxia-inducible factors 1α and 2α (HIF-1α and HIF-2α) and the PI3K/Akt/mTOR signaling pathway which provides fundamental pro-survival effects to leukemic cells. HIF-1α and HIF-2α interact with the “hypoxia-responsive elements” (HREs) of various genes (e.g., TGF-β, c-Kit, FGF-2, VEGF, and Notch-1) which may upregulate the expression of CXCR4 and CXCL12 on AML cells and ECs, thereby promoting LSC maintenance and chemotherapy resistance [139]. However, the role of hypoxia and downstream HIF-1α signaling in AML remains ambiguous, with published evidence for both promoting and repressive roles. In some studies, hematopoietic HIF-1α deletion supports AML progression in mice [140]. Similarly, combined deletion of HIF-1α and HIF-2α can accelerate AML initiation, but it is not essential for disease maintenance [141]. In contrast, other studies indicate that HIF-1α and HIF-2α support LSC survival by inducing p16 and p19 signaling and reducing ROS levels and endoplasmic reticulum stress, respectively [142,143].

Hypoxia-activated prodrugs (HAPs) are designed to specifically target cells in a hypoxic milieu. This targeting strategy is conceived by attaching a hypoxia-activated trigger to a chemotherapeutic agent. Under hypoxic conditions, the agent is released and activated while remaining intact in normal conditions and leaving non-hypoxic cells undamaged. TH-302 (Evofosfamide) is a HAP, which releases the DNA alkylating agent bromo-isophosphoramide mustard under hypoxic conditions. Primary human AML cells that were chemoresistant in oxygen-poor conditions became sensitive to Ara-C when treated with TH-302. The prodrug decreased HIF-1α expression, enhanced DNA double-strand breaks, and induced cell cycle arrest and apoptosis [144]. In AML xenografts, TH-302 can eliminate AML cells resistant to chemotherapy in hypoxic microenvironments and improve mice survival [145]. Clinical experience with TH-302 includes a phase I study in 39 patients with refractory AML [146]. All patients received standard chemotherapy in addition to TH-302. Although rapid early cytoreduction occurred in most patients, this was transient and not maintained until the next cycle and only two responded.

## 3. Mesenchymal Niche Remodeling in AML

BM stromal remodeling through altered differentiation of MSCs has emerged, besides well-described changes on ECs of BM vasculature [147], as a key factor in the development of myeloid malignancies. Although AML patient-derived MSCs do not differ from healthy donors in terms of phenotypic profile, alterations have been reported in specific subpopulation numbers, functions, or molecular profiles.

Osteoblastic lineage cells are a compartment of MSC progeny committed to the osteogenesis that comprises many intermediate stages of differentiation, including immature osteoprogenitors and mature bone-lining and bone-forming OBs.

In xenograft models, leukemia engrafted preferentially in association with microvascular domains in the BM [120,148]; especially for AML, blasts are also located in the bone endosteal surface [149,150] where they are found in close proximity to osteoblastic and osteoclastic cells. OBs are becoming interesting as pivotal regulators of leukemia outcomes. Ablation of OBs was shown to expedite leukemia progression in several mouse models of AML and ALL, with increased circulating blasts, higher tumor burden in BM and spleen, and reduced survival [151,152]. Maintenance of OB numbers during leukemia by pharmacological inhibition of the synthesis of duodenal serotonin through the use of the tryptophan–hydroxylase inhibitor LP533401, stimulated normal hematopoiesis, delayed disease engraftment, reduced tumor burden, and prolonged survival [152].

### 3.1. AML Induces Preosteoblastic-Rich Niche

Bone turnover is impaired in the leukemic niche. AML has been associated with the accumulation of OB-primed MSCs, which do not seem to be able to mature into OBs, correlating with decreased mineralized bone. Using an immunocompetent murine model of AML, Frisch et al. showed that osteoblastic cells were reduced and inhibited by leukemia, as measured by decreased levels of the bone formation marker, osteocalcin. Mineralized bone was also diminished. The cytokine CCL3 (macrophage inflammatory protein 1α; MIP-1α), known to be implicated in bone loss, was increased in AML blasts in mice and patients, suggesting that it may be related to the peculiar phenotype. Regarding the osteoclasts, there is an initial increase followed by a slight decline as the disease progresses to overt leukemia [151]. Another report found that AML cells seem to induce osteogenic differentiation but inhibit adipogenesis of MSCs. Here, induction of osteogenesis was ascribed to activation of Smad-1/5 signaling in MSCs by bone morphogenetic proteins (BMPs) derived from AML cells and to overexpression of connective tissue growth factor (CTGF) in MSCs, leading to an increase of pre-osteoblastic cells in the leukemic niche that enhances AML expansion [153]. We recently demonstrated that pediatric AML-MSCs, even when removed from their pathological environment, show an intrinsically abnormal differentiation pattern with altered osteogenesis. Using an in vivo system specific to assess the osteogenic potential, AML-MSCs exhibited a reduced mature bone formation capacity and developed an osteoprogenitor-rich niche with the presence of osterix+/osteocalcin—pre-OBs and osteocalcin+/Dentin matrix acid phosphoprotein 1 (DMP1)—immature osteocytes [154]. Additionally, Hanoun et al. showed that AML progression leads to a reduction of the sympathetic nervous system which causes an expansion of osteoblast-primed MSCs, which can contribute to AML progression [155].

MDS and AML patients show a reduction in OB numbers, reflecting a corresponding reduction in bone-formation rate without any changes in osteoclast numbers, and OB recovery correlates with better prognosis [152]. This is in agreement with clinical reports of osteopenia and osteoporosis related to a decrease in OB function described in newly diagnosed children or adults with acute leukemia [156,157]. In some of these studies, the reduction of disease burden following chemotherapy correlated with an increase in OB activity and bone mass [156,157].

**Figure 3:**
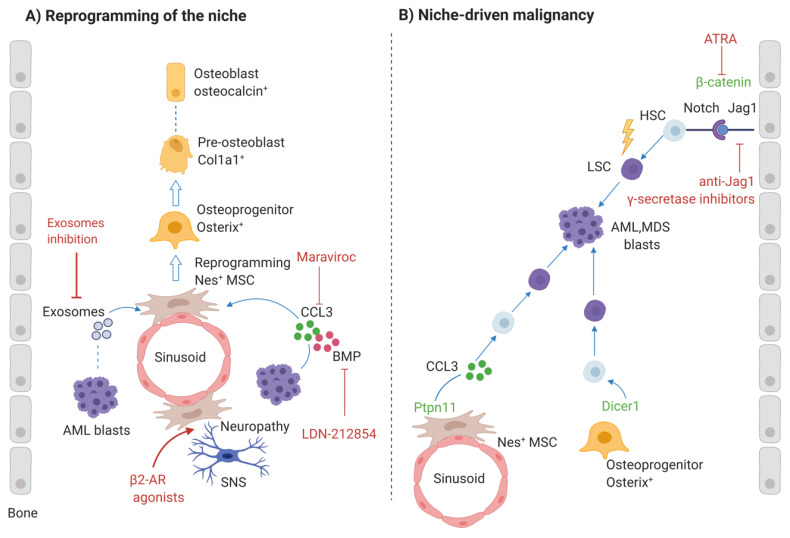
Osteogenic niche in leukemogenesis. (**A**) Leukemic cells reprogram BM niche into a self-reinforcing leukemic microenvironment. AML blasts induce osteogenic differentiation in MSCs through cell-to-cell contact and secretion of chemokine ligand 3 (CCL3) and bone morphogenetic proteins (BMP). Reduction of the sympathetic nervous system promotes the expansion of osteoblastic-primed MSCs, which can contribute to AML progression. Exosomes containing microRNAs (miRNAs) are secreted and uptaken by MSCs, impairing HSC function and favoring the proliferation of dysplastic cells. (**B**) Mutations in niche components have been associated with the initiation of myeloid malignancies. An activating mutation of β-catenin in osteoblasts induces AML in mice through upregulation of Jagged-1 expression. As a result, Notch1 signaling is activated in HSCs. A mutation in Dicer1 in Osterix+-osteoprogenitors deregulates HSCs that become dysplastic and eventually transform to AML. An activating mutation of the protein tyrosine phosphatase SHP2 (encoded by Ptpn11) in Nestin (Nes)^+^ MSCs leads to increased risk of leukemic transformation of HSCs via overproduction of CCL3. Potential therapeutic approaches targeting the MSCs remodeling and mutations are at pre-clinical stages (red). HSC: hematopoietic stem cell; LSC: leukemic stem cell; MSC: mesenchymal stromal cell; BMP: bone morphogenetic proteins; CCL3: C-C motif chemokine ligand 3; SNS: sympathetic nervous system; β2-AR: β2-adrenergic receptors; MDS: myelodysplastic syndrome; JAG1: Jagged1; ATRA: all-trans-retinoic acid. *This figure has been created with Biorender.com.*

Taken together, these observations indicate that AML cells induce and require an osteoprogenitor-rich niche for their expansion, but block MSC differentiation to produce mature OBs (Figure 3). It is tempting to speculate that inhibition of this “niche shaping” by leukemia cells, which alters the BM microenvironment to selectively support the malignant clone, would have a negative impact on leukemia progression.

In vitro experiments identified differentiating OBs as potent protectors of AML cells from various apoptosis-inducing agents, such as SDF-1 and standard chemotherapeutics Daunorubicin and Ara-C [158,159,160]. However, the mechanism(s) by which OBs induced chemoresistance of AML cells is not well-characterized. Pretreatment of differentiating OBs with histone deacetylase inhibitors (HDACi) Vorinostat and Panobinostat substantially disrupt their ability to protect AML cells from Ara-C, giving a rationale for clinical trials combining HDACi with Ara-C [161].

Blocking of CCL3 signaling can represent a further strategy to restore OB function, prime the BM for subsequent therapies to ablate leukemic cells and accelerate recovery of normal hematopoiesis. Following treatment with the small molecule Maraviroc which inhibits CCL3 binding to the chemokine receptor CCR5 presents on osteoblastic cells, the increase in MSC population is reversed and leukemic burden was decreased > 2-fold in the BM in a murine model of myelogenous leukemia [162]. Importantly, a long-term engrafting normal HSC population is maintained even in the complete absence of CCL3, suggesting that anti-CCL3 therapy would be well-tolerated by the normal hematopoietic system. However, due to the rapid metabolism and clearance of Maraviroc upon systemic administration, achieving therapeutically relevant doses in the BM niche is challenging. The use of novel marrow targeting nanoparticles approach to deliver Maraviroc is essential to maximize marrow selectivity and enhance Maraviroc-mediated CCL3 inhibition within BM [162].

Inhibition of BMP/CTGF-mediated signaling may also represent a novel therapeutic concept in AML to reduce leukemia growth. CTGF is a prognostic factor in ALL, and inhibition of its expression by an anti-CTGF antibody (FG-3019) led to increased survival of leukemia-bearing mice [163]. In vitro treatment of MSCs with BMP-type1 receptor-specific inhibitor LDN-212854 inhibits AML-induced pSmad1/5 upregulation and osteogenic differentiation [153].

#### 3.1.1. Role of AML-Induced Sympathetic Neuropathy

Neuropathy, which is characterized by decreased sympathetic nerve fibers and ensheathing Schwann cells, is observed in the BM of newly diagnosed AML patients [164]. Moreover, denervated mice that were transplanted with primary human AML exhibited higher levels of BM infiltration compared with controls, suggesting that neuropathy improves homing/engraftment of the AML cells [155].

The sympathetic nervous system is critical for MSC quiescence, OB differentiation and HSC maintenance [165]. In the MLL-AF9 AML model, AML caused sympathetic neuropathy in the BM which was shown to reinforce leukemia progression through the transformation of the HSC niche. The latter was associated with the reduction of arteriole-associated perycitic NG2+ cells that maintain HSCs and favor the expansion of leukemia-supportive Nestin+Leptin-receptor+ mesenchymal progenitors with increased commitment towards osteoblastic differentiation. These cells express lower levels of HSC-retention factors, including CXCL12, SCF, ANG1, and VCAM-1, and are unable to differentiate into mature OBs.

Although sympathetic neuropathy is observed both in myeloproliferative neoplasm (MPN) and AML, it has been demonstrated using experimental mice that defective β3-adrenergic signals are responsible for the phenotypes observed in MPN, whereas β2-adrenergic signals seem to be mainly involved in AML [155,166]. Thus, a potential therapeutic approach could be the use of receptors antagonists to overcome this β-adrenergic receptor damage. Although β3-adrenergic receptor agonists appear to be promising for the MPN therapy, the use of β2-adrenergic receptor agonists has provided contradictory results in AML.

#### 3.1.2. Role of Exosomes

Exosomes, cell-derived microvesicles of endocytic origin, may be another method that malignant cells use to reprogram the BM niche [167]. In AML patients, plasma exosome concentration was significantly increased compared to normal plasma and correlated with the disease burden, diminishing after conventional chemotherapy [168].

It was demonstrated in in vitro co-cultures that both primary AML cells and AML cell lines release exosomes containing coding and non-coding RNAs that can be uptaken by BM stromal cells, altering their secretion of growth factors and changing their proliferation and migration capability [169]. Moreover, AML-exosomes can contribute to the functional suppression of HPCs in the leukemic BM microenvironment, blocking the SCF and CXCL12 release by BM stromal cells [170].

Recently, Kumar et al. showed that AML cells use the secretion of exosomes as a means to adapt the niche to support leukemia growth and suppress normal hematopoietic development [171]. They demonstrated that AML-derived exosomes affect stromal cells and ECs in the BM niche. Using AML xenograft models, they have demonstrated that AML-derived exosomes caused changes in mice similar to those promoted by AML cells. Injection of AML-derived exosomes increase the number of mesenchymal stromal progenitors with blocked OBs differentiation in the BM niche and modulates gene expression in stromal cells. Genes involved in normal HSC sustainment and bone development (e.g., CXCL12, KITL and IGF1) were downregulated, whereas Dickkopf-1 (DKK1), a suppressor of hematopoiesis and osteogenesis, was upregulated.

Rab27a has been recently identified as one protein involved in exosome release and it represents a potential therapeutic target [172]. Rab27a knockdown blocks the ability of AML exosomes and AML cells to alter the BM composition of wild-type recipient mice. Specifically, Rab27a knockdown cancels the increase of SCA1+/CD146+ stromal cells, and the block of OB differentiation induced by DKK1 upregulation significantly prolongs the survival of wild-type recipient mice [171]. Disrupting exosome production and/or secretion in AML cells or directly targeting factors induced by AML-derived exosomes, such as DKK1, may provide new perspectives to avoid AML-induced transformations of BM niche.

### 3.2. Role of Altered Osteogenic Niche in the Pathogenesis of AML

Under certain conditions, osteolineage cells have been demonstrated to contribute to the alteration of HSCs, triggering the formation of leukemia-initiating cells. Signals from the microenvironment may indeed select target cells for subsequent transforming events and, therefore, they may represent candidate therapeutic targets in both treatment and prevention strategies (Figure 3). Specifically, it has been demonstrated that the genetic manipulation of specific stromal cell subsets can drive leukemogenesis in different mouse models.

In the first model, the conditional deletion of Dicer1 in mouse osteoprogenitors impairs osteogenic differentiation and causes myelodysplasia with sporadic transformation to AML [173]. The loss of Dicer1 is associated with reduced expression of the ribosome maturation factor encoded by the *SBDS* (Shwachman-Bodian-Diamond syndrome) gene mutated in Schwachman-Diamond syndrome, a human congenital BM failure with known leukemia predisposition [174].

Subsequently, it has been reported that mutations activating β-catenin in OBs in mice induce myelodysplasia, rapidly progressing to AML [175]. These investigators also found that activated β-catenin signaling is present in OBs of one-third of MDS and AML patients and it is the most active pathway in stromal cells of MDS patients, suggesting that it may sustain dysplastic hematopoiesis and progression to MDS and AML also in humans. Therefore, targeting this pathway may represent a new therapeutic approach for this subgroup of patients. Treatment of leukemic mice expressing constitutively active β-catenin in their OBs with all-trans-retinoic acid (ATRA) inhibited β-catenin signaling, improved anemia and thrombocytopenia, decreased the amount of blasts in BM and blood, and prolonged overall survival [176]. Moreover, it has been shown that activated β-catenin leads to the development of AML through upregulation of Jagged1 expression in OBs and subsequent activation of Notch signaling in hematopoietic cells [175]. Inhibition of osteoblastic Notch signaling by Jagged1 deletion or pharmacologic treatment with γ-secretase inhibitors prevents AML development in mice. Furthermore, blocking Jagged1/Notch signaling between OBs and HSCs using an anti-JAG1 antibody efficiently treated OB-induced MDS/AML in mice [177]. The Kousteni’s group attributed this niche-induced leukemogenesis to the oncogenic role of FoxO1 in OBs that interacts with β-catenin and upregulates Notch ligand expression [178]. This observation suggests targeting FoxO signaling in OBs may be helpful for patients with constitutive activating β-catenin mutation.

Finally, activating mutations of the Tyrosine phosphatase SHP-2 (encoded by Ptpn11 gene) in MSCs and osteoprogenitors, already found in Noonan syndrome and associated with an increased risk progression to leukemia, induce juvenile myelomonocytic leukemia-like myeloproliferative neoplasm in mice through the overproduction of chemokine CCL3 [179]. This study defines CCL3 as a potential therapeutic target for leukemia progression control in patients with Noonan syndrome.

While these findings in mice offer direct evidence for OB-induced leukemogenesis and although some observations in mouse models have been linked to human diseases, it remains unclear whether alterations to the microenvironment can drive leukemia in humans. Emerging reports of donor cell leukemia in patients receiving allogeneic transplantation (only 1–5% of all post-transplant leukemia relapses) seem to suggest an oncogenic role of the microenvironment that can lead to secondary malignancy also in humans [180].

### 3.3. Adipocytes-Rich Niche and Fatty Acid Metabolism

Adipocytes derive from MSC differentiation are prevalent in the BM stroma and their number augment with age. MSCs from AML patients have a higher propensity to differentiate into adipocytes, and the interactions between adipocytes and AML blasts in the BM niche support their survival and proliferation [181]. We recently demonstrated using an innovative in vivo model of humanized hematopoietic niche that AML-MSCs-derived ossicles contained a significantly increased fraction occupied by adipocytes [154].

AML blasts modulate adipocyte metabolism, inducing lipolysis of triglyceride to fatty acid (FA) through induction of hormone-sensitive lipase and growth differentiation factor 15 (GDF15) release [182,183]. In these conditions, AML blasts shift their metabolism toward fatty acid β-oxidation (FAO), obtaining the energy required for leukemic growth and proliferation. These AML-adipocyte interactions have been linked to chemotherapeutic resistance [184,185]. Obesity is associated with poor clinical outcome in leukemic patients and AML marrow in remission has less adipocytes content than non-remission marrow [186,187].

Increasing attention is being paid on metabolic alterations in AML as potential therapeutic targets and encouraging results have been achieved in preclinical AML models using several inhibitors of FA metabolism. Pharmacological inhibition of FAO by carnitine palmitoyltransferase 1a (CPT1a) inhibitor was reported to decrease the pro-survival effects of adipocytes on AML. Moreover, Lee and colleagues identified a novel FAO inhibitor derived from the avocado fruit, avocatin B, to be a potent inhibitor of AML survival and proliferation [188]. Shafat et al. proposed that fatty acid binding-protein 4 (FABP4) is important for the transfer of lipids from adipocytes to AML and its expression is increased in adipocytes and AML when in co-culture [183]. FABP4 inhibition using FABP4 short hairpin RNA or a small molecule inhibitor blocks AML proliferation on adipocyte layers and increases survival of an AML model. Pharmacological inhibition or lentiviral knockdown of FABP4 in the adipocytes showed a significant inhibitory effect on AML survival in co-culture experiments. Always in the context of FA transfer, a target candidate for inhibition is CD36, a FA transporter. Ye et al. have demonstrated that LSCs can be divided into two distinct CD36− and CD36+ subpopulations, with the latter displaying an increased FAO activity and drug resistance profile [184].

Nevertheless, FAO inhibition triggers the activation of bypass metabolic pathways to enhance survival of AML in the BM microenvironment. This indicates a possible limited efficacy of these metabolic inhibitors when used as single agents. In AML cells co-cultured with BM adipocytes, FAO inhibition with avocatin B increased glucose uptake and switched to glycolysis [185]. Alternatively, FAO inhibition combined with conventional chemotherapy or targeted therapy has a synergistic effect in the eradication of BM-resident, chemoresistant AML cells. FAO inhibition by avocatin B is highly synergistic with Ara-C by increasing ROS production and apoptosis in AML cells co-cultured with BM adipocytes [185]. Similarly, Farge et al. showed that the FAO inhibitor Etomoxir increased sensitivity to Ara-C of resistant AML cells [189].

Adipocytes contribute to the AML protection also by other pro-AML survival factors in addition to the FA release. BM adipocytes have been shown to secrete high levels of pro-inflammatory cytokines in the context of leukemia, which may have an impact on leukemia cell migration and survival [184].

## 4. Emerging Strategies to Improve the Modeling of Human AML Niche

### 4.1. In Vitro AML Models

The first in vitro AML niche models were 2D cultures based on the Dexter system where an established feeder stromal cell layer allowed for the prolonged viability and stemness of hematopoietic cells. van Gosliga and colleagues developed and extensively tested the first long-term co-culture system for the ex vivo maintenance of primary AML cells based on an immortalized mouse MSC line as the feeder layer [190]. To overcome the species incompatibility, Ito at al. tested a co-culture model in which primary human BM stroma was used as a feeder layer supporting AML survival for 4–6 weeks [191]. The Bonnet laboratory further developed the 2D co-culture system and compared the drug response of AML cells maintained ex vivo to that of cells transplanted in immunodeficient mice [192]. The 2D co-culture system has also been applied for high-throughput drug screening [193]. Moreover, new small molecules, such as StemRegenin 1 (SR1) and UM729, have been identified, supporting the ex vivo maintenance of undifferentiated AML cells otherwise rapidly lost in culture [194]. Overall, these models hold promise because they are cheap, allow tight control of the system components, and are easy to manipulate. However, they are insufficient to mimic the in vivo situation of the 3D trabecular bone microenvironment.

To overcome this limitation, numerous 3D models have been developed using a range of biomaterials as scaffolds designed with properties comparable to those of the in vivo BM, including pore size and surface cell-adhesive features, to allow the establishment of cell–niche associations [195]. Efforts were made to add more complexity to these models through the use of new synthetic materials with easy-to-modulate properties and the addition of further extracellular matrix components. Bray et al. generated a 3D co-culture with human umbilical vein endothelial cells (HUVEC), MSCs, and AML to investigate the impact of vascular niche in AML and they found that this scaffold supports the resistance of leukemic cells against chemotherapy [196]. Furthermore, Kotha and colleagues also developed an artificial in vitro vascular marrow and showed that patient-derived AML cells increase their adhesion and migration in response to distinct fibroblasts [197].

The BM microenvironment is also strictly regulated by the concentration of soluble factors, oxygen levels, and the mechanical stress applied by blood flow. The use of bioreactors can then allow for modeling the niche situation more closely. Rödling et al. developed a bioreactor system for perfusion of 3D scaffolds mimicking the BM in vivo and demonstrated the importance of perfusion during drug treatment as results are different with and without perfusion [198].

The most advanced BM niche model incorporates an in vivo implantation stage in a perfused 3D co-culture ex vivo. One of these hybrid approaches (also known as engineered BM or bone marrow on-a-chip) was developed by the Ingber laboratory by first engineering new bone in vivo, explanting it whole and maintaining it in vitro with culture medium in a microfluidic device [199]. This enables mouse MSCs and circulating HSCs to populate a biomimetic scaffold, leading to the establishment of a physiological hematopoietic niche that closely resembles the natural BM with well-developed and/or distinguished endosteal and perivascular niches, multiple stromal cell types, and extracellular matrix. Unfortunately, none of the hybrid models have yet been tested for their ability to support AML cells, retain LSC functionality, or reproduce chemotherapy resistance mediated by the leukemic niche. To determine the true potential of an in vitro model, its ability to replicate the in vivo behavior of AML cells is still essential.

### 4.2. In Vivo AML Models

Patient-derived xenograft (PDX) mouse models are currently the gold standard for studying the development of human leukemia and testing new therapies. Notably, engraftment and expansion of human AML in vivo remain challenging as a substantial number of samples fail to engraft the most optimized host mice, particularly less aggressive malignancies [200]. It may be that some subtypes of AML have low progenitor cell frequency, or some samples may be particularly sensitive to the lack of a cell type in the mouse BM or a factor that is poorly or not at all cross-reactive between mice and humans. NSG transgenic mice expressing human factors such as SCF, GM-SCF, IL-3, and thrombopoietin (TPO) have been developed and allowed better engraftment of primary AML samples [201]. However, long-term exposure to high levels of these human factors caused the reduction of HSC self-renewal [202]. More recently, MISTRG mice were developed, whereby macrophage colony-stimulating factor (M-CSF), IL-3, GM-CSF, and TPO were knocked-in in their respective mouse loci, together with a BAC transgene encoding for human SIRPα, consenting a more physiological level of human cytokines production [203]. These mice supported the development of innate immune cells in vivo and robust engraftment of otherwise difficult-to-engraft primary AML samples [204]. Yet, in these human cytokine mice essential human niche-specific factors might still be lacking. The interaction of AML cells with specific human niche components or immune cells might be critically important. However, despite the ongoing efforts, the achievement of a fully functional human immune system (including the lymphoid system) in mice is still far away.

Due to the mismatch between human leukemia cells and the mouse BM niche, traditional xenograft models are insufficient to study AML microenvironment-targeted therapies, and to overcome this limitation, several humanized xenograft mouse models are developing. Xenotransplantation approaches are rapidly evolving toward injecting human hematopoietic cells into immunodeficient mice implanted with ectopic humanized BM niches. The first study of AML engraftment in humanized microenvironment was reported by Vaiselbuh et al., who developed an ectopic humanized niche by implanting subcutaneously in NOD/SCID mice polyurethane scaffolds coated with human MSCs. The scaffolds formed structures mimicking the human BM niche, including mineralized bone-matrix, OBs, and stromal cells, as well as the appropriate tissue vascularization. Primary AML cells injected directly in pre-implanted scaffold or intravenously in mice, successfully engrafted in the ectopic niche [205]. Further studies demonstrated that AML samples non-engrafting in mouse BM were able to efficiently engraft in the humanized microenvironment [206,207,208]. These studies have developed various protocols which differ for carrier material scaffold (ceramic, gelatin or hydroxyapatite-based scaffolds), human stromal cell types used (BM-derived MSCs co-injected or not with human endothelial colony-forming cells or total human BM cells), and experimental time frames (from 12 to 34 weeks) [209,210]. Importantly, ossicles maintained the clonal heterogeneity in xenografted AML cells and their LSC self-renewal capacity was retained as demonstrated by serial transplantation assay [211].

Finally, Battula et al. developed a different approach called “human bone implant” that consists of subcutaneous transplants in NSG mice of fragments from freshly collected human BM biopsies using matrigel as a carrier [153]. The implanted human BM tissue undergoes vascularization and bone restoration in mice, providing a functional human BM microenvironment capable of supporting the human leukemia engraftment.

These models are useful tools for the functional assessment of niche contribution, as well as for the development of novel anti-leukemic modalities aimed at modifying the AML microenvironment. Moreover, these new approaches, which also allow genetic manipulation of human niche components, can likely help in better understanding the role of factors critical for leukemia engraftment/progression. A pilot study demonstrated that the deletion of HIF-1α in human MSCs impaired AML engraftment in BM organoids by decreasing SDF-1α expression [212].

Of note, current 3D models use MSCs isolated from healthy donors, which are molecularly and functionally different from disease-exposed ones. The use of patient-derived niche components may further improve these models and help unravel the role of intercellular communication in disease-relevant systems. We recently reported an AML stromal niche model obtained using MSCs derived from AML patients [154]. Questions remain regarding the specific role of the vasculature, which is exclusive of mouse origin, as well as the cytokines supplied by the mouse system to the human cells engrafted within these humanized scaffolds. Nevertheless, this new approach may provide an important tool to generate patient-specific BM niches in mice that can be used to elucidate the role of the human AML microenvironment and to carry out preclinical studies for the development of new targeted therapies. Continuous development efforts are, however, required to improve their effectiveness and physiological relevance.

## 5. Conclusions

The final goal of AML research is to find new strategies to effectively eradicate LSCs to prevent therapy relapse and, ultimately, offer a cure to patients.

Targeting the altered adhesion and homing of AML cells to the niche is a promising approach to prevent relapse, as to displace LSCs from their protective niche may allow for their full eradication. Development of new approaches to counteracting the soluble factors that promote malignant niche formation also has therapeutic potential. Moreover, finding new systems to directly target the specific features of the malignant BM niche, including aberrant MSC differentiation, also may be helpful at a therapeutic level. Thus, these types of AML niche-targeted strategies may represent a useful approach to complement chemotherapy.

A main challenge is represented by the dual role of the microenvironment in regulating normal and malignant hematopoiesis. Therapies targeting the tumor microenvironment must simultaneously eliminate chemo-resistant AML cells and preserve/re-establish normal hematopoiesis.

Our understanding of niche contributions to AML has hugely increased over the last decades, however, many questions remain unsolved; in particular, whether the phenotypes and molecular mechanisms identified in mouse models are maintained and therapeutically relevant in the human disease. The use of human leukemia samples to understanding AML niche biology is not allowed as diagnoses are made on BM aspirates that disrupt BM architecture.

Moreover, most of the pre-clinical research to study drug effects on AML progression is done in xenograft mice. In the case of therapies targeting the AML microenvironment, this may represent an important limitation in the translation of animal model results to human patients due to the mismatch between human leukemia cells and the mouse BM niche.

Functional assessment of niche contribution to AML and pre-clinical testing of new niche-targeted therapies require the establishment of disease-relevant model systems. Researchers’ efforts should be focused to build bone/BM organoids systems that aim to closely recapitulate the human malignant BM niche complexity.
